# Hyperphosphorylation of the Group A Streptococcal Control of Virulence Regulator Increases Promoter Occupancy Specifically at Virulence Factor-Encoding Genes

**DOI:** 10.1128/jb.00118-23

**Published:** 2023-06-08

**Authors:** Nicola Horstmann, Chau Nguyen Tran, Anthony R. Flores, Samuel A. Shelburne

**Affiliations:** a Department of Infectious Diseases, Infection Control and Employee Health, The University of Texas MD Anderson Cancer Center, Houston, Texas, USA; b Division of Infectious Diseases, Department of Pediatrics, McGovern Medical School at UTHealth Houston, Houston, Texas, USA; c Department of Genomic Medicine, The University of Texas MD Anderson Cancer Center, Houston, Texas, USA; University of Illinois Chicago

**Keywords:** ChIP-seq, CovRS, DNA binding, *Streptococcus pyogenes*, two-component regulatory systems

## Abstract

The control of virulence two-component gene regulatory system (CovRS) is critical to the pathogenesis of many medically important streptococci. In *emm1* group A streptococci (GAS), CovR directly binds the promoters of numerous GAS virulence factor-encoding genes. Elimination of CovS phosphatase activity increases CovR phosphorylation (CovR~P) levels and abrogates GAS virulence. Given the *emm* type-specific diversity of CovRS function, in this study we used chromatin immunoprecipitation sequencing (ChIP-seq) to define global CovR DNA occupancy in the wild-type *emm3* strain MGAS10870 (medium CovR~P) and its CovS phosphatase-negative derivative 10870-CovS-T284A (high CovR~P). In the wild-type *emm3* strain, 89% of the previously identified *emm1* CovR binding sites present in the *emm3* genome were also enriched; additionally, we ascertained unique CovR binding, primarily to genes in mobile genetic elements and other sites of interstrain chromosomal differences. Elimination of CovS phosphatase activity specifically increased CovR occupancy at the promoters of a broad array of CovR repressed virulence factor-encoding genes, including those encoding the key GAS regulator Mga and M protein. However, a limited number of promoters had augmented enrichment at low CovR~P levels. Differential motif searches using sequences enriched at high versus low CovR~P levels revealed two distinct binding patterns. At high CovR~P, a pseudopalindromic AT-rich consensus sequence (WTWTTATAAWAAAAWNATDA) consistent with CovR binding as a dimer was determined. Conversely, sequences specifically enriched at low CovR~P contained isolated ATTARA motifs suggesting an interaction with a monomer. These data extend understanding of global CovR DNA occupancy beyond *emm1* GAS and provide a mechanism for previous observations regarding hypovirulence induced by CovS phosphatase abrogation.

**IMPORTANCE** Given its key role in pathogenesis of Gram-positive bacteria, CovR is one of the most important members of the OmpR/PhoB family of transcriptional regulators. Herein we extend recent GAS CovR global binding analyses done in *emm1* to a non-*emm1* strain, which is important considering the known inter-*emm*-type heterogeneity in GAS CovRS function. Our data provide mechanistic understanding for variation in CovRS function between *emm* types and the profound hypovirulence of CovS phosphatase-negative strains in addition to indicating differential targeting by phosphorylated and nonphosphorylated CovR isoforms at specific CovR binding sites. These findings advance knowledge regarding how a key bacterial virulence regulator impacts pathogenesis and add to the growing appreciation of the function of nonphosphorylated OmpR/PhoB family members.

## INTRODUCTION

The external environment poses challenges to bacteria, such as toxic molecules, but also provides critical substances, including nutrients and key enzymatic cofactors (1–3). Thus, a rapid and efficient response to environmental cues is critical to bacterial pathophysiology (4, 5). Given their lack of a nuclear compartment, prokaryotes can readily transfer exterior signals to DNA effector proteins using two-component gene regulatory systems (TCS) comprised of a membrane-embedded histidine kinase which controls the phosphorylation status and hence activity of a cognate response regulator (6, 7). Although extensively studied for many years, new discoveries about bacterial TCS continue to accumulate, and these systems are considered potential novel therapeutic targets (8–11).

The control of virulence TCS CovRS, also known as CsrRS (for capsule synthesis regulator/sensor) (12), consists of the histidine kinase CovS and the response regulator CovR (13). The CovRS system is highly conserved among beta-hemolytic streptococci and is a key virulence effector for the major human pathogens group A and group B Streptococcus (GAS and GBS) (14–18). Additionally, CovR homologues are present in a wide range of major Gram-positive pathogens (e.g., ArlR in Staphylococcus aureus) (19, 20).

*emm1* GAS is commonly studied as a result of being the most common *emm* type causing both invasive and noninvasive GAS infections (21, 22). Similarly, *emm3* GAS is another well-investigated GAS *emm* type given that it produces a disproportionately high percentage of invasive disease (23–25). Under laboratory conditions, in *emm1* GAS, CovS primarily serves to phosphorylate CovR such that phosphorylated CovR (CovR~P) accounts for ~75% of total CovR, with the remainder being nonphosphorylated (26). CovS can also lower the CovR~P/CovR ratio through its phosphatase activity, which is augmented in the presence of the human antimicrobial peptide LL37 (26, 27). In *emm3* GAS, a conserved mutation in the regulator of CovR (RocA) causes CovR~P to be only ~40% of total CovR (25, 28). CovS threonine 284 is critical to CovS phosphatase activity such that a T284A alteration increases CovR~P levels to ~80% in both *emm1* and *emm3* GAS and abolishes GAS responsiveness to LL37 (26, 27). In both *ex vivo* human and mouse models of infections, we previously found that *emm1* and *emm3* CovS-T284A strains had a marked virulence defect (27). Although well studied in both GAS and GBS for some 20 years (12, 14, 15, 29–31), only recently have CovR global binding analyses been published, including two analyses in distinct *emm1* GAS strains and one analysis in GBS (32–34). These studies have shown that CovR directly binds to the promoters of a diverse array of virulence factor-encoding genes in both GAS and GBS, thus establishing CovR as a paradigm for investigating direct bacterial virulence factor regulation.

Given the broad genetic diversity of GAS and known heterogeneity in CovRS function for different *emm* types (35–38), herein we detail the global CovR binding characteristics of an *emm3* strain. Additionally, we sought to gain insight into the dramatic virulence impact of CovR hyperphosphorylation by assessing global CovR binding in a CovS-T284A isoallelic strain. These data delineate both conserved and specific aspects of the CovR regulon in *emm1* and *emm3* GAS strains and show that abrogating CovS phosphatase activity markedly augments CovR occupancy at a broad repertoire of GAS virulence factor-encoding genes.

## RESULTS

### CovR global binding analysis in the *emm3* strain MGAS10780.

We analyzed CovR DNA binding in the *emm3* strain MGAS10870, which has previously been fully sequenced and long used as a representative isolate of the GAS *emm3* population (23). To this end, we performed chromatin immunoprecipitation sequencing (ChIP-seq) and included the strain 10870 Δ*covR* as a negative control. Using the parameters detailed in Materials and Methods, we identified 83 significantly enriched DNA regions in the immediate vicinity of 82 genes (Table 1), of which 44 (54%) are considered part of the CovR regulon. The majority of the enriched DNA regions (59/83 [71%]) were located within promoter regions (39), particularly for genes considered part of the CovR regulon (43/44 [98%]) (27, 40). These data affirm the idea that direct CovR binding in gene promoter regions is the major mechanism by which CovR impacts gene expression (33). The sole exception to this was the streptolysin S (*sag*) operon (41), in which CovR binding was observed at the 3′ but not 5′ end.

**TABLE 1:** CovR DNA binding sites identified in this study with comparison to binding sites previously identified in MGAS2221 (M1) [32, 33]

Gene	SpyM3 no.	Promoter location	Avg RPKL value in:		MGAS 2221 no.	Binding in M1 [32, 33]
			MGAS10870	CovS-T284A		
*dnaA*	_0001	No	838	1,844	_0001	Wt^*b*^, Mg^2+^, LL37
*hpt/ftsH*	_0011/12	No	1,432	631	_0012/13	Wt, E281A, LL37
	_0013	Yes	(563)	1,353	_0012/13	Wt, E281A, LL37
*rpoB*	_0075	Yes	1,508	720	_0128	Wt, E281A, LL37, Mg^2+^,
*nra*	_0097	Yes	4,001	2,954	Absent	
*hypo*	_0105	Yes	1,691	2,334	_0159	Wt, E281A, LL37, Mg^2+^,
*nga*	_0128	Yes	678	1,322	_0183	Wt, Mg^2+^,
*hypo*	_0131	Yes	12,136	25,243	_0187	Wt, E281A, LL37, Mg^2+^,
*hypo*	_0132	Yes	6,565	28,492	_0188	Wt, E281A, LL37, Mg^2+^,
*ulaA* (*sgaT*)	_0134	Yes	699	503	_0191	Wt, E281A, LL37, Mg^2+^
*hypo*	_0154	Yes	953	1,792	_0221	Wt
*speG*	_0155	No	1,078	(701)	_0222	E281A, LL37, Mg^2+^
*rivR*	_0158	Yes	683	1,011	_0225	LL37, Mg^2+^
*rgpG*	_0207	No	812	(219)	_1646	E281A, LL37
*oppA/dacA*	_0214	Yes	968	3,427	_1638/9	Wt, E281A, LL37, Mg^2+^
*hypo*	_0231	Yes	743	981	_0328	LL37, Mg^2+^
*braB*	_0236	Yes	2,745	604	_0333	Wt, E281A, LL37, Mg^2+^
*dahA*	_0243	Yes	2,143	4,672	_0340	Wt, Mg^2+^
*covR*	_0244	Yes	1,563	828	_0341	Wt, E281A, LL37, Mg^2+^
*scpA/B*	_0267/8	No	1,157	(391)	_0365	E281A, LL37
*prtS*	_0298	Yes	5,433	34,282	_0397	Wt, E281A, LL37, Mg^2+^
*spyA*	_0304/5	Yes	5,583	23,728	_0403/4	Wt, Mg^2+^
*hypo*	_0307	Yes	851	3,447	_0406	Wt, E281A, LL37, Mg^2+^
*hypo*	_0343	Yes	1,460	4263	_0450	Mg^2+^
*mfs transp/licT*	_0402/3	Yes	1,606	(497)	_0516/7	Wt, E281A, LL37
*riboflavin*	_0425/6	Yes	1,659	2,571	_0538/9	Wt, E281A, LL37, Mg^2+^
*ftsW*	_0431	Yes	986	(325)	_0544	E281A, LL37
*sagA*	_0480	Yes	(863)	2,067	_0589	No
*sagI*	_0488	No	653	1,319	__0606	Wt, E281A, LL37, Mg^2+^
*rpsU/mscl*	_0516/7	No	1,520	(527)	_0633/4	LL37
*trxB*	_0575	No	1,713	1,319	_0691	Wt, E281A, LL37, Mg^2+^
*mac-1*	_0583	Yes	767	3,198	_0700	Wt, E281A, LL37, Mg^2+^
*engB*	_0605	No	840	(630)	_0720	LL37, Mg^2+^
*bcaT*	_0626	Yes	1,425	542	_0742	Wt, E281A, LL37, Mg^2+^
*hypo*	_0669	Yes	947	(455)	_0788	LL37
*sclB*	_0738	Yes	1,505	7,344	_0802	Wt, E281A, Mg^2+^
*potD*	_0767/8	No	1,250	850	_0849	E281A, LL37
*regulator*	_0827/8	Yes	757	(447)	_0915	LL37
*ffh*	_0841	No	659	(283)	_0930	LL37, Mg^2+^
	_0869	No	(441)	1,116	_0993	Wt, E281A, LL37, Mg^2+^
*cfa*	_0905	Yes	1,106	1,991	_0993	Wt, E281A, LL37, Mg^2+^
*grab*	_1032	Yes	707	2,149	_1119	Wt, E281A, LL37, Mg^2+^
*queA*	_1066	No	1,029	(432)	_1153	E281A, LL37, Mg^2+^
*hypo*	_1069/70	Yes	1,394	(641)	_1157/8	LL37, Mg^2+^
*kup/deaD.1*	_1078/9	Yes	793	(182)	_1166	LL37, Mg^2+^
*mf4*	_1095	Yes	1,072	13,882	Not in M1	
*coaD*	_1188	No	1,208	(447)	_1275	Wt, E281A
*asnA*	_1190	Yes	634	(178)	_1277	LL37
*slaA*	_1204	Yes	797	1,307	Not in M1	
*trxB*	_1288	Yes	1,830	(677)	_1314	Wt, E281A, LL37, Mg^2+^
*speA*	_1300	Yes	(566)	1,075	_0996	Wt, Mg^2+^
*phage*	_1311	No	1,208	1,196	Not in M1	
*phage*	_1312	No	1,050	914	Not in M1	
*comFA*	_1361	No	863	(492)	_1341	E281A, LL37
*sdn*	_1409	Yes	1,604	1,588	Not in M1	
*hypo*	_1473	Yes	944	1,860	_1401	Mg^2+^
*hypo*	_1477/8	No	1,299	(338)	_1405/6	E281A, LL37, Mg^2+^
*esterase*	_1493	Yes	890	2,804	_1422	Wt, Mg^2+^
*manL*	_1511	Yes	978	(362)	_1496	E281A, LL37
	_1534/5	NO	985	(443)	_1518	LL37
*hypo*	_1537	NO	777	(412)	_1521	LL37
*codY*	_1544/5	YES	2,865	6,790	_1527	Wt, E281A, LL37, Mg^2+^
*aminotransf*	_1545	Yes	720	(238)	_1528	E281A, LL37
*ABC transp*	_1550	Yes	682	1,432	_1533	Wt, LL37, Mg^2+^
*Isp.2*	_1562	Yes	(653)	1,502	_1552	Wt, Mg^2+^
*endoS*	_1568	Yes	876	2,803	_1552	Wt, Mg^2+^
*hypo/mutY*	_1583/4	Yes	1,906	6,534	_1567/8	Yes
*XRE family pr*	_1605/6	Yes	1,068	2,286	_1589	Wt, LL37, Mg^2+^
*hypo*	_1626	Yes	1,046	2,741	_1610	Mg^2+^
*ska*	_1698	Yes	8,823	52,579	_0254	Wt, E281A, LL37, Mg^2+^
*sclA*	_1703	Yes	19,928	61,179	_0251	Wt, E281A, Mg^2+^
*dppA*	_1718	Yes	1,202	(433)	_0236	Wt, E281A, LL37, Mg^2+^
*dppE/*	_1722/3	No	2,539	4,557	_0232	Wt, E281A, LL37, Mg^2+^
*scpA*	_1726	Yes	1,049	2,811	_1697	Wt, Mg^2+^
*emm3*	_1727	Yes	622	2,015	_0668	E281A, Mg^2+^
*mga*	_1728	Yes	1,222	3,956	_1701	Wt, E281A, LL37, Mg^2+^
*grm*	_1739	Yes	1,343	5,359	_1711	Wt, Mg^2+^
*mf*	_1745	Yes	760	874	_1717	LL37, Mg^2+^
*ahpC*	_1770	Yes	2,428	2,671	_1743	Wt, E281A, LL37, Mg^2+^
*treP*	_1786	Yes	787	(299)	_1759	LL37
*hypo*	_1795	Yes	2,945	21,086	_1771	Wt, E281A
*hypo*	_1795	No	2,218	(909)	_1769	Wt, E281A, LL37, Mg^2+^
*hypo*	_1843	Yes	2,351	6,888	_1817	Wt, E281A, LL37, Mg^2+^
*zinc protease*	_1850	No^*a*^	1,869	6,467	_1824	Wt, E281A, LL37, Mg^2+^
*hasA*	_1851	Yes	3,690	30,434	_1825	Wt, E281A, LL37, Mg^2+^
*guaA/B*	_1856/7	Yes	1,690	3,279	_1830/1	Wt, E281A, LL37, Mg^2+^
*trsA*	_1858	No	2,555	945	_1832	Wt, E281A, LL37, Mg^2+^
*spd1*	spyM18_0779	Yes	628	798	_1832	Wt, E281A, LL37, Mg^2+^

a Binding influences expression of *hasA*.

b Wt, wild type.

We used reads per kilobase length (RPKL), a normalized metric of amount of immunoprecipitated DNA, to evaluate the strength of CovR DNA interaction (33). Strikingly, the top-scoring binding sites were almost exclusively observed in the promoters of known CovR-regulated virulence genes, e.g., *sclA*, which encodes a cell surface collagen binding protein (42); *ska*, which encodes the critical plasminogen-activating protein streptokinase (43); *prtS*, which encodes an interleukin 8 (IL-8)-degrading enzyme (44); and *hasA*, which encodes the first gene of the hyaluronic acid capsule-encoding operon (45). Importantly, *speB* (encoding cysteine protease) was the only CovR-regulated virulence factor-encoding gene for which no significant DNA enrichment was observed (46). We and others have previously identified repression of pilus genes in *emm3* GAS and *in vitro* binding of recombinant CovR to the intergenic region between the *nra* gene (40, 47), which encodes a transcriptional activator of the pilus genes (48), and *cpa*, the first gene of the pilus-encoding operon (49). Consistent with direct CovR regulation of *nra* and *cpa*, we observed a dual peak in this region (see Fig. S1 in the supplemental material), with a high RPKL peak detected in the 5′ region of the *nra* gene and a smaller peak situated in the *nra*/*cpa* intergenic region (Fig. S1).

### Comparison of *emm1* and *emm3* wild-type CovR binding.

**FIG 1:**
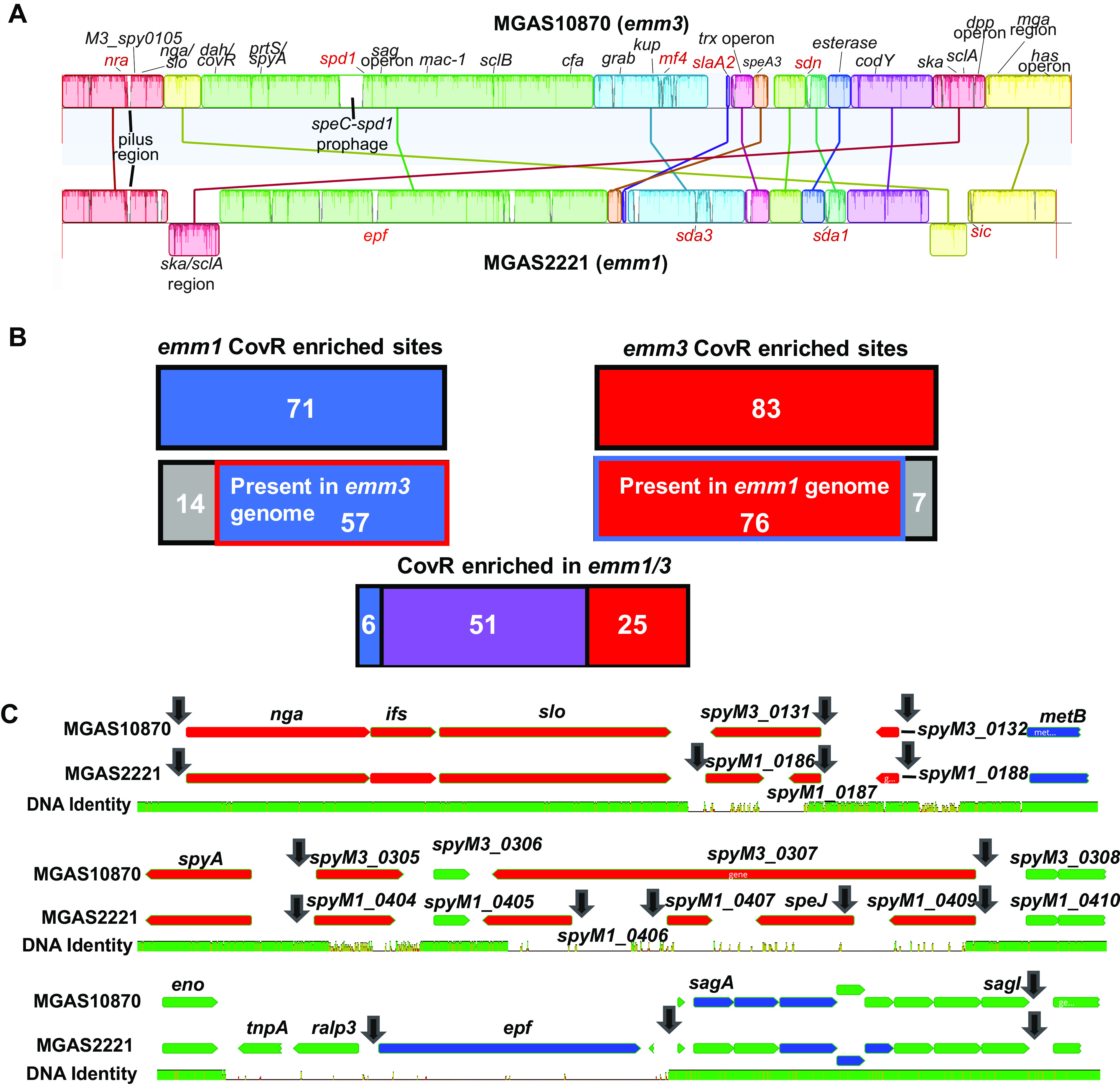
(A) Genome alignment of MGAS10870 (top) and MGAS2221 (bottom) generated with Mauve software, with colors indicating locally colinear blocks shared within the two respective genomes. Lines indicate rearrangement of genes or regions. Selected directly CovR-regulated genes are labeled in black (present in both strains) and red (present in only one *emm* type). (B) Comparison of CovR enriched regions in the *emm1* and *emm3* strains. (C) Genomic sequence variation in the context of the *nga*-*slo* operon (top), the *spyA* region (middle), and the *sag* operon (bottom) between MGAS10870 and MGAS2221. Genes are depicted as horizontal arrows in the direction of transcription and labeled with genes repressed at high CovR~P levels shaded red, genes activated at high CovR~P levels shaded in blue, and those unaffected by changing CovR~P shaded green based on data from reference 27. The line below indicates the sequence identity between the two strains. Black vertical arrows show CovR-enriched sites.

We next sought to compare our findings in the *emm3* strain MGAS10870 to our data generated in the *emm1* strain MGAS2221 using the same antibody and approach (Table 1). The two genomes are generally highly similar (average nucleotide identity of 98.8% by the DNAdiff function in MUMmer [50]), although MGAS2221 has an ~50-kbp area of recombination relative to strain MGAS10870, which contains the directly CovR-regulated genes *ska* and *sclA* (Fig. 1A).

Of the 71 CovR DNA-enriched regions (±200 bp from the peak) in the *emm1* strain MGAS2221, 57 (80%) are present at ≥90% nucleotide homology in the MGAS10870 genome (Fig. 1B). Conversely, of the 83 CovR-enriched sites in MGAS10870, 76 (92%) exist at ≥90% homology in the MGAS2221 genome. Half of the *emm*-type-specific peaks (11/21) were in the promoters of genes encoding CovR-regulated virulence factors such as DNases (e.g., *sda1* in *emm1*), phospholipases (e.g., *slaA2* in *emm3*), and complement inhibitors (*sic* in *emm1*) (see red labels in Fig. 1A). Further, we observed genomic sequence variation impacting CovR binding immediately downstream of the *nga-slo* operon (which encodes the key cytotoxin streptolysin O [51]), the *spyA* region (which encodes an ADP-ribosyltransferase as well as several hypothetical proteins [52]), and upstream of the streptolysin S-encoding *sag* operon (41) (Fig. 1C). Thus, these data reveal strain-to-strain variation as to the presence of directly CovR-regulated virulence factor-encoding genes.

Next we analyzed CovR-mediated enrichment of DNA sites that are present in both genomes (Fig. 1B). Fifty-one of the 57 (89%) CovR-binding regions previously identified in strain MGAS2221 that share homologous sequences with strain MGAS10870 were also significantly enriched in MGAS10870, whereas 6 (11%) were not. Of these six, only one, namely, *sagA*, was in the promoter region of a CovR-regulated virulence factor. In contrast, only 51 (67%) of the 76 CovR-bound regions in MGAS10870 which were present in strain MGAS2221 were also significantly enriched in the latter. About half of the remaining 25 sites (12 [48%]) were situated in promoter regions, but only 2 were in the promoters of CovR-regulated genes, namely, *rivR* and *spd*. Both exhibited small peaks that did not meet our significance criteria in our previous *emm1* data (33). Interestingly, more than half of these sites (13 out of 25 [52%]) were previously enriched in the CovS kinase activity-deficient strain 2221-CovS-E281A (low CovR~P) (33), raising the question of whether these sites may be specifically bound by unphosphorylated CovR. Consistent with the idea that unphosphorylated CovR preferentially binds nonpromoter DNA (32), only 4 of these 13 sites were in promoter regions.

To assess whether the same *cis*-regulatory elements were targeted by CovR in the *emm1* and *emm3* strains, we compared the locations of the 51 CovR-enriched DNA regions observed in both MGAS2221 and MGAS10870. The median deviation from average peak location between the two strains was only 13 nucleotides, with a 25th to 75th percentile of 7 to 33 bp (see example of peak variance for *ska* in Fig. S2A). For comparison, the median peak variance between biological replicates was 13 bp (25th to 75th percentiles of 7 to 22) and 9 (2 to 23) for MGAS10870 and MGAS2221, respectively. There was no statistical difference (analysis of variance [ANOVA] > 0.05) between the interstrain versus intersample (i.e., biologic replicates) variance (Fig. S2B). Thus, our ChIP-seq data generated highly reproducible peaks that covered very similar regions in *emm1* and *emm3* strains, suggesting that CovR was likely binding the same *cis*-regulatory elements for both strains.

### Impact of inactivating CovS phosphatase activity on global CovR DNA interaction.

**FIG 2:**
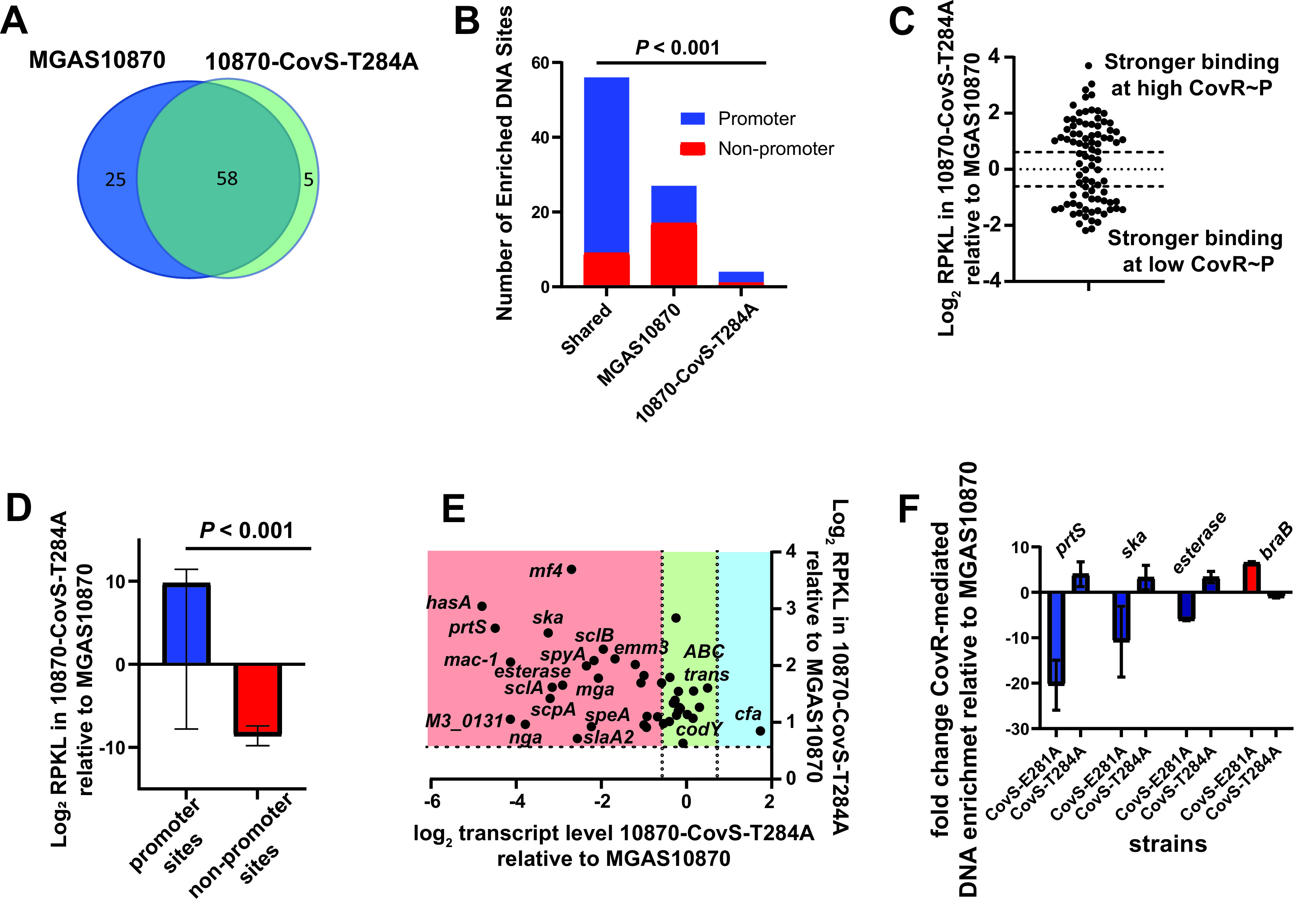
Influence of CovS phosphatase activity on CovR DNA binding and transcription regulation. (A) Venn diagram depicting the number of joint (turquoise) and individual (blue and green) binding loci between strain MGAS10870 and 10870-CovS-T284A. (B) Number of enriched DNA loci separated by location in the indicated strains. (C) Bimodal distribution of CovR binding in 10870-CovS-T284A relative to MGAS10870 at CovR-enriched promoters. (D) Graph depicting distinct CovR~P-dependent binding at promoter versus nonpromoter loci. (E) Correlation of CovR~P dependent DNA enrichment and transcript levels. Dashed lines indicate 1.5-fold difference in transcript levels. (F) Fold change enrichment of indicated promoters in the low (E281A)-CovR~P and high (T284A)-CovR~P strains relative to the wild type as measured by SYBR qPCR. Note the opposite CovR~P-dependent enrichment for promoters of selected GAS virulence factors (blue) and *braB* (red).

We next sought to gain insights into how increasing CovR~P impacts CovR DNA interaction by performing ChIP-seq in strain 10870-CovS-T284A (~75% CovR~P levels). Although CovR phosphorylation is thought to be critical to CovR DNA interaction (13), we identified fewer CovR-enriched DNA sites in strain 10870-CovS-T284A than in the wild type (63 versus 83 [Table 1]). The two isoallelic strains shared 58 sites, of which 48 (83%) were in promoter regions (Fig. 2A and B) and included such well-established CovR directly regulated virulence factor-encoding genes as *hasA*, *prtS*, and *mac-1*, also known as *ideS*, which encodes an immunoglobulin-degrading enzyme (53). Of the 25 DNA-enriched sites exclusively present in the low CovR~P strain MGAS10870, only 12 (48%) were in promoter areas, and none of these genes encode known CovRS-regulated virulence factors. However, five of these genes have been previously identified as CovR regulated, namely, *dppA*, which is the first gene in an operon encoding a dipeptide transport system (54); *spyM3_0402*, which encodes a transporter; the first gene in the *trx* operon (55); *asnA*, which encodes a protein involved in asparagine metabolism; and *treP*, which encodes a protein involved in trehalose transport. In contrast, of the five CovR binding sites uniquely identified in the high-CovR~P strain 10870-CovS-T284A, four were in promoter regions (Table 1), including the first gene of the streptolysin S-encoding operon (*sagA*). The ratio of promoter relative to nonpromoter enrichment was significantly lower for sites identified only in strain MGAS10870 compared to sites identified only in 10870-CovS-T284A or sites shared between strains (*P* < 0.001 by Fisher’s exact test [Fig. 2B]).

### CovR hyperphosphorylation specifically increases DNA enrichment at CovRS-repressed virulence factor-encoding genes.

Comparison of the RPKL values for individual sites within MGAS10870 and 10870-CovS-T284A revealed a nonnormal distribution with two clusters, i.e., those with significantly higher RPKL values in strain 10870-CovS-T284A and those with higher values in strain MGAS10870 (Fig. 2C). These data correspond well with the similar bimodal distribution we detected for the previously published RPKL ratios between strain MGAS2221 and 2221-CovS-E281A (Fig. S3A) (33). Moreover, we observed a strong correlation between RPKL ratios for the high-CovR~P/low-CovR~P strains between the two *emm* types (*R*^2^ = 0.74; *P* < 0.001 [Fig. S3B]). These data indicate that the impact of CovR~P variation on CovR binding is independent of the *emm* type. Moreover, sites within a promoter had higher RPKL levels in strain 10870-CovS-T284A, whereas nonpromoter sites tended to have higher RPKL values in strain MGAS10870 (*P* < 0.001 by Mann-Whitney test [Fig. 2D]), consistent with previous data suggesting that unphosphorylated CovR preferentially targets nonpromoter loci (32).

Next, we related the RPKL ratios between strains MGAS10870 and 10870-CovS-T284A to previously published transcript-level differences between the two strains (27). We identified 39 promoters which had ≥1.5-fold enrichment in strain 10870-CovS-T284A relative to MGAS10870. Of these 38 genes, the T284A mutation resulted in a decrease, no significant change, and increase in transcript levels for 24, 13, and 1 gene (63%, 34%, and 3%), respectively. Importantly, nearly all CovR-regulated virulence factor-encoding genes showed increased CovR binding and lower transcript levels in the phosphatase-negative strains (Fig. 2E, red shading). Conversely, none of the 13 genes with similar transcript levels between the two strains encode a known GAS virulence factor but rather encode proteins involved in metabolism and regulatory function (Fig. 2E, green shading). Finally, the single gene with both higher CovR binding and transcript levels in the CovS-T284A strain, indicating direct activation by CovR, was *cfa* (Fig. 2E, blue shading), which encodes a cyclic AMP factor (56). At 16 promoters, CovR bound stronger in strain MGAS10870 (i.e., lower CovR~P), and only one of these genes, namely, *dppA*, had decreased transcript levels in the phosphatase-deficient strain. None of these 16 genes encode a known virulence factor; rather, they encode proteins involved in metabolic functions and regulators such as CovR itself. We included strain 10870-CovS-E281A, with a CovR~P level of ~20%, to validate the distinct influence of CovR~P status (lowest in CovS-E281A, medium in the wild type, and highest in CovS-T284A) on CovR DNA enrichment using SYBR quantitative PCR (qPCR). As expected, CovR enrichment increased with increased CovR~P levels for the tested GAS virulence factor genes *prtS*, *ska*, and *esterase*, whereas *braB*, which encodes a branched-chain amino acid transporter, showed the opposite pattern (Fig. 2F). Taken together, our findings show that inactivation of CovR phosphatase activity results in specific increased CovR binding and transcriptional repression at the promoters of major CovR-regulated virulence factor-encoding genes.

### Binding site location relative to transcriptional start site affects CovR~P impact on gene transcript levels.

As shown by the green shading in Fig. 2E, there were several CovR-bound promoters which evidenced variation in CovR binding but not in transcript levels when CovR~P levels were increased. These genes are not typically considered part of the CovR regulon, so we sought to gain insight into this observation by locating the CovR binding site within the respective promoters. For genes whose transcript levels were significantly impacted by changes in CovR~P levels along with stronger CovR-mediated enrichment (i.e., red shaded area in Fig. 2E), there was a median distance of 25 bp from the transcriptional start site (TSS) to the peak of CovR binding (Fig. S4). Conversely, for genes that evidenced higher CovR~P promoter binding without significant impact of CovR~P on transcript levels, the median distance from peak CovR binding to the TSS was 105 bp (*P* = 0.01 by Mann-Whitney U test) (Fig. S4). Given the known interaction of CovR~P with RNA polymerase, these data suggest that binding of CovR close to the transcription initiation complex contributes to transcript-level variation in response to changing CovR~P levels (57).

### Identification of distinct binding motifs targeted at low versus high CovR~P levels.

**FIG 3:**
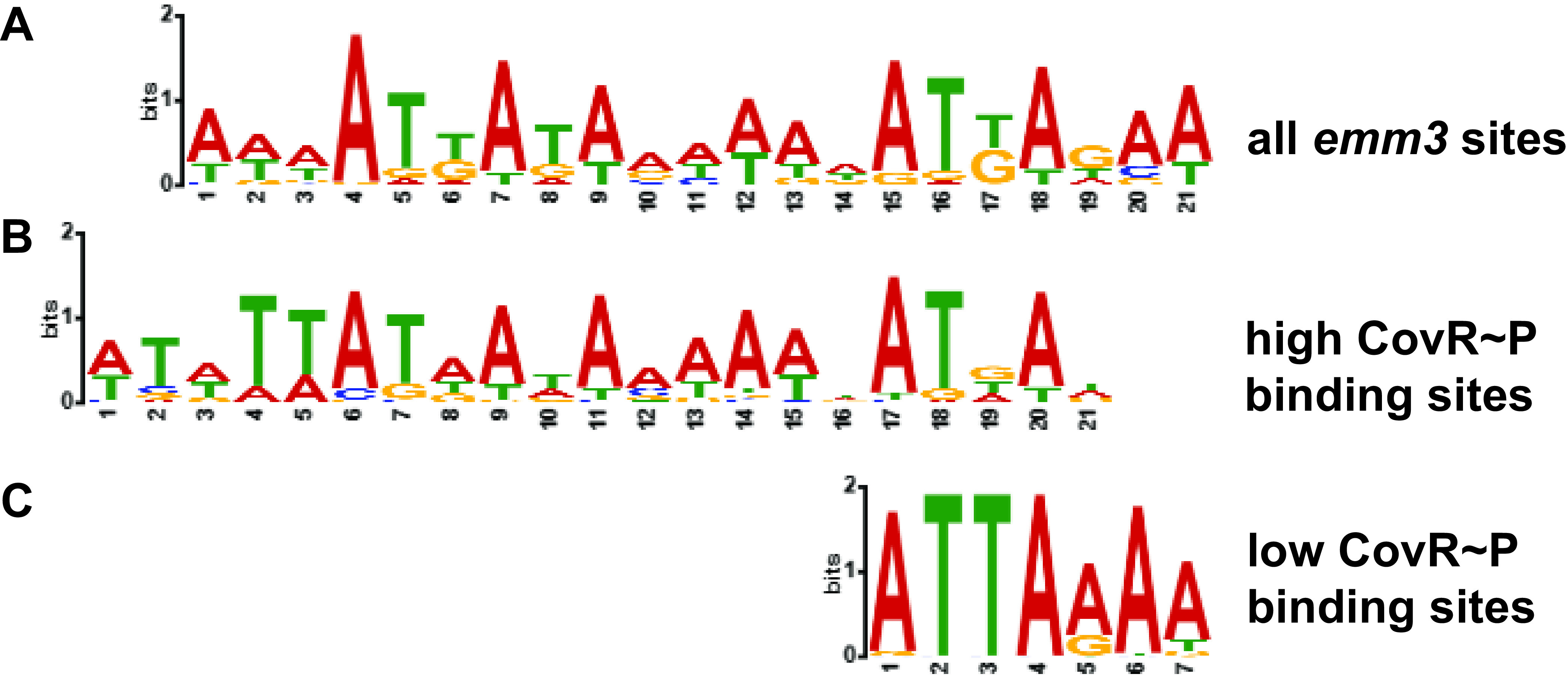
CovR binding motifs identified by MEME using different cohorts of promoter sequences as indicated.

We have previously identified a putative CovR binding motif in the *emm1* strain MGAS2221 by restricting our search to only sites with high RPKL values and established CovR regulation (33). In this study, we included all *emm3* CovR binding regions in our MEME searches and identified a highly similar AT-rich motif (Fig. 3A). Since our analyses of CovR binding characteristics between low and high CovR~P strains of both serotypes suggested varied impacts of altering CovR~P levels for distinct promoters, we also assayed the two promoter groups separately to discern possible differences in *cis*-regulatory elements between sites with preferential high versus low CovR~P binding. The identified motif for high CovR~P binding with a significant *P* value of e^−43^ resembled the one identified for all *emm3* sites with a slight enrichment for TT at its 5′ end (Fig. 3B) which has previously been identified as important for CovR binding at *hasA* and *sagA* promoters (29, 58). Interestingly, when we included only CovR sites that evidenced more enrichment at lower CovR~P levels, we identified a much shorter motif that consisted of a single strongly conserved ATTARA sequence with no conserved surrounding nucleotide structure (*P* = e^−10^ [Fig. 3C]).

### Insights into genes with atypical pattern of CovR regulation.

**FIG 4:**
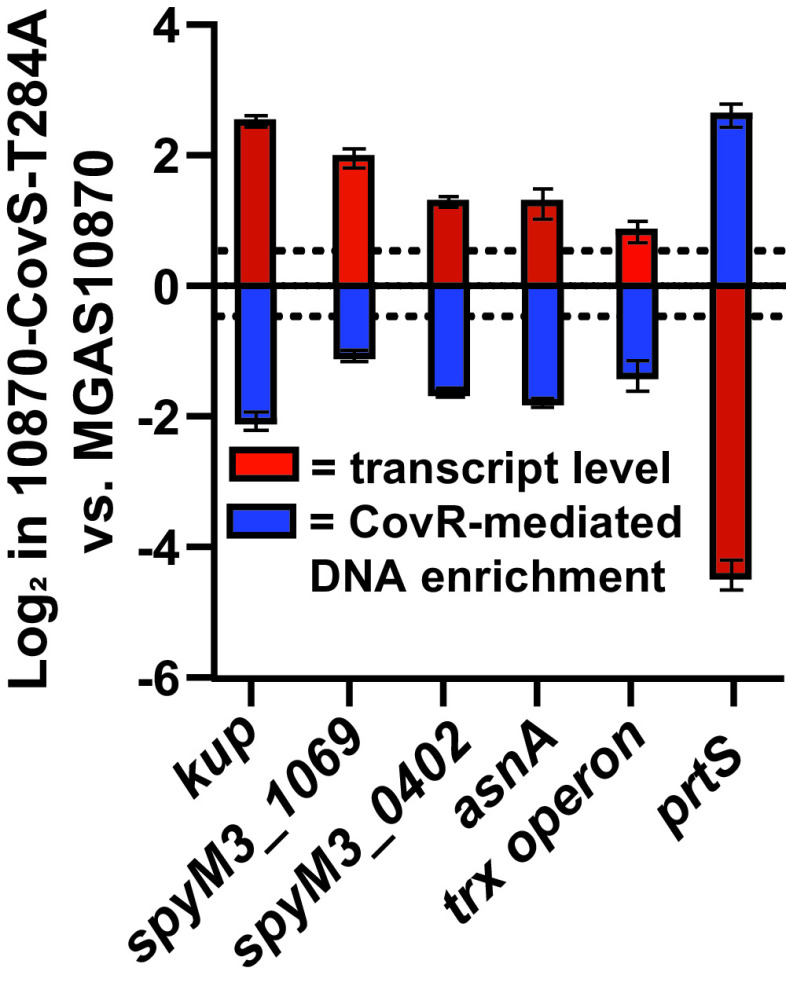
CovR~P influence on CovR DNA binding and gene transcript levels for directly CovR regulated genes with atypical CovR regulation pattern. Data shown are log_2_ mean values ± standard deviations for CovR DNA binding (blue) and transcript level (red) in strain 10870-CovS-T284A relative to MGAS10870 for selected CovR directly regulated genes. The first five genes have atypical CovR regulation pattern (i.e., higher transcript levels at higher CovR~P levels), whereas *prtS*, a typical CovR~P-repressed virulence factor-encoding gene, is included for reference purposes. Dashed lines indicate 1.5-fold cutoffs.

One of the enigmatic observations in CovRS-related studies is that CovR and CovS inactivation can have opposing effects on the transcript levels of certain genes even though CovS is thought to act only via CovR (59). For strain MGAS10870, we identified five CovR directly repressed genes (*kup*, *spyM3_1069*, *spyM3_0402*, *asnA*, and the first gene of the *trx* operon) with increased transcript levels at higher CovR~P levels (27, 47). It has been hypothesized that this transcript level pattern could be ascribed to better binding of unphosphorylated CovR than CovR~P, resulting in relieved repression both in the absence of CovR and at higher CovR~P (60). Consistent with this hypothesis, we observed increased CovR binding for each of these five genes in MGAS10870 compared to 10870-CovS-T284A (Fig. 4, with *prtS* included as a typical CovR~P-repressed gene). *speB* is considered a paradigm of the CovR-repressed, CovS-activated phenotype (59), but similar to our findings in MGAS2221, we did not observe significant CovR enrichment in the vicinity of the *speB* gene for either strain MGAS10870 or 10870-CovS-T284A (33). Overall, our data are consistent with the hypothesis that a subset of CovR-regulated genes are repressed by unphosphorylated CovR.

## DISCUSSION

Two-component signaling pathways are critical to bacterial pathogenesis and thus remain a highly active area of investigation across a broad array of major human pathogens (61–63). Herein, we delineate the global binding characteristics of the critical streptococcal response regulator CovR, in both a wild-type *emm3* GAS and a CovS phosphatase-inactive strain. Together with recently published data from *emm1* strains (32, 33), these data expand knowledge regarding CovR global DNA binding activity, provide mechanistic insights into strain-to-strain variation in the CovRS regulon, and demonstrate that elimination of CovS phosphatase activity specifically increases CovR binding and hence repression of a critical array of GAS virulence factor-encoding genes.

An important finding of both our *emm3* and previous *emm1* data from our lab and Finn et al. (32, 33) is that CovR binds to promoter regions of nearly all GAS virulence factor-encoding genes. With some 1,000 TSS in GAS (39), the selective targeting of promoter elements of such a diverse array of GAS virulence factor-encoding genes by CovR is remarkable, in particular given that many CovR directly regulated genes are present in nonconserved areas of the genome acquired via horizontal gene transfer, such as those encoding a diverse variety of DNases (Fig. 1A) (32, 33, 64). Mazzuoli et al. hypothesized that the intergenic regions of newly acquired GBS virulence factor-encoding genes rapidly evolve to facilitate CovR regulation (34). Consistent with a similar mechanism being at play for GAS, we observed minimal CovR binding to mobile genetic element DNA outside the promoters of virulence factor-encoding genes. In addition to CovR binding distinct areas of mobile genetic elements that were different between *emm1* and *emm3* GAS (64), we also identified numerous CovR directly regulated genes in highly variable chromosomal locations not typically associated with mobile genetic elements, such as downstream of the *nga-slo* operon, the *spyA* region, and the *sag* operon area (Fig. 1C). Thus, strain-to-strain variation in the presence of direct CovR targets provides potential mechanisms for the long-recognized strain-dependent impact of CovS inactivation on GAS virulence (16, 35, 65).

By comparing the intermediate CovR~P *emm3* strain MGAS10870 with its high-CovR~P isoallelic mutant 10870-CovS-T284A, we were able to discern the effect of CovS phosphatase activity on specifically directing CovR binding close to the TSSs of a diverse array of virulence factor-encoding genes. *In vitro* work has previously shown that CovR~P interacts with RNA polymerase to increase CovR DNA binding affinity at the *has* promoter (57). Thus, these data suggest that the hypovirulence induced by CovS phosphatase inactivation (27) likely results from accumulation of CovR~P near TSSs, perhaps through augmented interaction with RNA polymerase, and subsequent silencing of the transcriptional process. Importantly, CovR was strongly enriched at the promoters of both *emm3* and *mga* in strain 10870-CovS-T284A, which is consistent with our previous observation of reduced *emm3* and *mga* transcript levels in CovS-T284A strains (27). *emm* and *mga* have been found to be crucial for the emergence of hypervirulent GAS strains with CovRS mutations (66, 67), and accordingly, such hypervirulent strains rarely emerge from strains with the CovS-T284A background (27). Morreall et al. showed that the generation of mutant Escherichia coli strains under stress requires sufficient survival of the parental strain to allow for generation of mutants (68). Thus, we hypothesize that the direct repression of the genes *emm3* and *mga*, along with other virulence factor-encoding genes, engendered by the CovS-T284A mutation does not allow for enough bacterial survival while interacting with human immune components to permit generation of mutated strains with altered CovRS status. Inasmuch as targeting of bacterial TCS is an area of potential antimicrobial development (10, 69, 70), the impact of the CovS-T284 mutation on generation of mutated strains is critical to avoid the emergence of hypervirulent GAS when attempting to abrogate GAS virulence by targeting CovS phosphatase activity.

Although the vast majority of GAS virulence factor-encoding genes showed higher CovR binding and transcript level repression in strain CovS-T284A, there was a subset of genes which exhibited the opposite pattern, namely, higher CovR binding and lower transcript levels in the low CovR~P strain MGAS10870. Given that previous studies have shown these genes to be CovR repressed (47), the most parsimonious explanation is that unphosphorylated CovR binds better to these promoters and hence represses more relative to CovR~P. Thus, our data add to the growing appreciation that the canonical view of an OmpR/PhoB family regulator being active only when phosphorylated and dimerized is likely an oversimplification (71). Similarly, in Streptococcus mutans, where CovS is absent, CovR regulatory function is independent of phosphorylation status (72). The potential activity of a CovR monomer is further supported by our finding of a single ATTARA CovR DNA binding motif (as originally identified by June Scott’s group [29, 58, 73]) for genes which demonstrated higher CovR enrichment at lower CovR~P levels as opposed to the longer, nearly symmetrical tandem DNA binding motif present in promoters enriched at higher CovR~P. The residual activity of unphosphorylated CovR may account for the observation that hypervirulent GAS strains predominantly contain mutations leading to CovS inactivation rather than complete abrogation of CovR function (37, 74, 75).

In summary, herein we present CovR ChIP-seq analyses for *emm3* strains with both low and high CovR~P levels and compare these data to those recently obtained using a similar approach in *emm1* GAS. Our findings extend knowledge regarding the critical function of CovRS and provide a rationale for targeting CovS phosphatase activity in order to maintain CovR promoter occupancy, thereby silencing production of nearly the entire GAS virulence factor repertoire.

## MATERIALS AND METHODS

### Bacterial strains, media, and growth conditions.

MGAS10870 is a fully sequenced clinical isolate of an *emm3* GAS strain. The strain has a wild-type *covR/S* sequence (the CovR amino acid sequence is identical to that of MGAS2221, but there is a V332I amino acid exchange in CovS compared to CovS in MGAS2221 which does not affect CovRS function [26]) and thus produces SpeB. For a detailed analysis of SpeB expression in MGAS10870, see reference 76. However, MGAS10780 does not express a full-length RocA protein due to a frameshift mutation that generates a premature stop codon. Consequently, MGAS10870 has a medium CovR~P level (~45%). The isoallelic isolates M3-CovS-E281A and M3-CovS-T284A have low (~20%) and high (~75%) CovR~P due to mutations that affect CovS kinase and phosphatase activity, respectively (27). GAS strains were grown without agitation in Todd-Hewitt broth supplemented with 0.2% yeast (THY medium) at 37°C under 5% CO_2_.

### ChIP and sequencing.

Three biological replicates of strains MGAS10870, M3-CovS-T284A, and 10870 Δ*covR* (control), respectively, were chromatin immunoprecipitated using polyclonal antibody directed against the N-terminal part of CovR as described previously (33). Briefly, GAS strains were grown in 40 mL of THY medium to mid-exponential phase (optical density [OD] ~ 0.45), proteins were cross-linked to DNA with 1% formaldehyde, and cells were harvested by centrifugation, flash-frozen, and stored at −80°C. The fixed cell pellets were resuspended in 1 mL of ice-cold lysis buffer, and the lysates were sonicated for 15 cycles (30 s on/30 s off) at 4°C in 1.5-mL Bioruptor tubes (Diagenode) in a Diagenode Bioruptor Plus machine set at high power, to shear DNA to fragments with lengths of 200 and 400 bp. The cleared supernatant was collected to use for chromatin immunoprecipitation (ChIP) (950 μL) or input DNA (50 μL), respectively. CovR-bound DNA fragments were immunoprecipitated overnight at 4°C using Dynabeads protein G (Invitrogen) precoated with anti-CovR_ND_ antibody. After several washing steps, the complex was eluted with 50 μL of elution buffer to obtain the ChIP DNA (output) samples. Proteins and RNA in both input and ChIP samples were degraded using RNase A and proteinase K, cross-linking was reversed by incubation at 65°C overnight, and the DNA was purified using SPRI beads (AMPure XP; Beckman Coulter) on a magnetic stand. The DNA concentration was determined on a Qubit machine 4.0 (Invitrogen) following the Qubit manual for high-sensitivity DNA, and DNA fragment size distribution was assessed using Agilent D1000 screen tape on an Agilent 2200 TapeStation system. ChIP sequencing was performed in the Advanced Technology Genomics Core (ATGC) Facility at MD Anderson Cancer Center as described previously (33) with Illumina-compatible indexed libraries prepared from 2 to 10 ng of sheared ChIP or input DNA. Equimolar quantities of the indexed libraries were multiplexed with 8 libraries per pool and sequenced on an Illumina NextSeq500 sequencer using the high output 75-nucleotide (nt) single-read flow cell format.

### Analysis of ChIP-seq data.

Raw sequencing reads (~30 million to 35 million [M] reads per replicate/input sample) were quality filtered, trimmed, and mapped to the reference genome MGAS10870 using CLC Genomics Workbench (v 21; Qiagen). Peaks representing potential CovR binding were identified using the Transcription Factor ChIP-seq module of CLC Genomics Workbench as described previously (33). Peak shape scores and RPKL values were plotted against each other and peaks were manually inspected to identify a reliable threshold for statistically significant enrichment of DNA. Peaks with a peak shape score of >30 and RPKL value of >500 in at least two of the samples were called as statistically significant. A gene was associated with an enriched DNA region if the peak center was within 200 bp of the promoter or the open reading frame of the gene.

### Correlation of CovR binding and influence on gene expression.

Binding data derived from ChIP sequencing in this study were correlated with previously generated transcriptome data (RNA-seq) of the *emm3*-type strains MGAS10870 and 10870-CovS-T284A (27). The CovR regulon was defined as gene with a ≥2-fold difference in transcript levels between MGAS10870 and 10870 Δ*covR* or between CovR strains with different CovR~P levels (M3-CovR-D53A versus M3-CovS-T284A). For analogue comparative analyses in *emm1* GAS, we used the ChIP and RNA-seq data of strains MGAS2221 and 2221-CovS-E281A generated in the studies described in references 33 and 27.

### Motif search.

A search for a CovR DNA binding motif within the sequences bound by CovR *in vivo* was conducted using the MEME-ChIP program implemented in Multiple Em for Motif Elicitation (MEME), suite 5.1.1. Different cohorts of sequences covering the average peak ± 200 bp were included in the searches. An E value of <0.05 was considered required for statistical significance of the motif.

### SYBR qRT-PCR.

Enrichment of selected promoters in the ChIP samples derived from strains MGAS10870, M3-CovS-E281A, and M3-CovS-T284A relative to input DNA was assessed by SYBR quantitative real-time PCR (qRT-PCR) on a StepOne Plus machine (Applied Biosystems) using Ssoadvanced universal SYBR green supermix (Bio-Rad) and the primers listed in Table S1 in the supplemental material. Fold enrichment of promoter DNA was normalized to enrichment of *ldh* (not regulated by CovR). Measurements were done in duplicate with at least two biological samples.

### Data availability.

ChIP-seq data have been released in the GEO database under accession number GSE230870.
