# Supplementary material for: Hyperphosphorylation of the Group A Streptococcal Control of Virulence Regulator Increases Promoter Occupancy Specifically at Virulence Factor-Encoding Genes
Source: J Bacteriol. 2023 Jun 8;205(6):e00118-23. doi: 10.1128/jb.00118-23 (PMC10294628; doi:10.1128/jb.00118-23)
Supplement: Supplemental file 5 — Table S1 and legends of Fig. S1 to S4. Download jb.00118-23-s0001.docx, DOCX file, 0.02 MB [file jb.00118-23-s0001.docx]

**SUPPLEMENTAL FIGURE LEGENDS**

**Fig. S1. CovR binding in the *nra*/*cpa* region of MGAS10870.** Genes are represented by solid, horizontal arrows in the direction of transcription and labeled below. Depth of mapped reads generated by ChIP-seq is shown in blue with peaks indicating CovR binding sites. Y-axis depicts 1000x depth of read mapping.

**Fig. S2. Peak variance between *emm1* and *emm3* samples.** (*A*) Peak variance between *emm1* (blue) and *emm3* (green) samples at the *ska* promoter. The colored lines indicate the range of peaks within the three replicates with the average peak center marked as arrow, respectively. The transcription start site (TSS) and the beginning of the open reading frame (ORF) are labelled. (*B*) Graph shows the peak variance between samples in number of nucleotides for each CovR bound site in the *emm1* and *emm3* strains (i.e. variance between biological replicates), respectively, as well as the average peak variance between *emm* types.

**Fig. S3. Comparison of impact of changing CovR~P levels on CovR DNA binding in *emm1* and *emm3* GAS.** (*A*) RPKL ratios of CovR binding sites in MGAS2221 (high CovR~P) relative to 2221-CovS-E281A (low CovR~P). (*B*) Comparison of RPKL ratios between high and low CovR~P strains in *emm1* (y-axis) and *emm3* (x-axis). R^2^ value refers to correlation with P indicated value.

**Fig. S4.** **Correlation of CovR~P DNA binding location with transcriptional regulation.** Promoters evidencing significantly increased CovR-mediated DNA enrichment in strain 10870-CovS-T284A vs. MGAS10870 were divided into those which did (left) or did not (right) have significantly different transcript levels between the two strains. Individual dots represent the distance (in bps) from the peak of CovR-DNA binding to the TSS taken from [62]. P value refers to Mann-Whitney test between the two groups.

**SUPPLEMENTAL TABLES**

**Table S1 Primers used in this study**

| **Name** | **Sequence** | **Description** |
| --- | --- | --- |
| esterase_SYBR_fwd | GTTGCATAACGTCACCTTC | SYBR qPCR |
| esterase_SYBR_rev | CAAGGGTATCTCCTTAATG | SYBR qPCR |
| prtS_SYBR_fwd | GTT ACA AGG CTT TCG TTT AAC | SYBR qPCR |
| prtS_SYBR_rev | CCT GAT ACC CTC CTA AAT GT | SYBR qPCR |
| ska_SYBR_fwd | AT TAT CAT GAC ATT ATC ATT AAG | SYBR qPCR |
| ska_SYBR_rev | AGA AAC CTC CTA AAA GTT AAG | SYBR qPCR |
| braB_SYBR_fwd | TGTGCTAAGAATTTTCAGTCAAT | SYBR qPCR |
| braB_SYBR_rev | CATGAATCCAATGACGATATATACA | SYBR qPCR |
